# Predicting the risk of postoperative gastrointestinal bleeding in patients with Type A aortic dissection based on an interpretable machine learning model

**DOI:** 10.3389/fmed.2025.1554579

**Published:** 2025-05-19

**Authors:** Lin Li, Xing Yang, Wei Guo, Wenxian Wu, Meixia Guo, Huanhuan Li, Xueyan Wang, Siyu Che

**Affiliations:** ^1^Department of Nursing, Shanxi Bethune Hospital, Shanxi Academy of Medical Sciences, Third Hospital of Shanxi Medical University, Tongji Shanxi Hospital, Taiyuan, China; ^2^First Clinical Medical School, Shanxi Medical University, Taiyuan, China

**Keywords:** Type A aortic dissection, gastrointestinal bleeding, machine learning, prediction model, the SHapley Additive exPlanation

## Abstract

**Background:**

Gastrointestinal bleeding (GIB) is a common complication following Type A aortic dissection (TAAD) surgery, significantly impacting prognosis and increasing mortality risk. This study developed and validated a predictive model based on machine learning (ML) algorithms to enable early and precise assessment of postoperative GIB risk in TAAD patients.

**Methods:**

Medical records of patients who underwent TAAD surgery at Shanxi Bethune Hospital from January 2019 to September 2024 were retrospectively collected. Predictors were screened using LASSO regression, and four ML algorithms—Random Forest (RF), K-nearest neighbor (KNN), Support Vector Machines (SVM), and Decision Tree (DT)—were employed to construct models for predicting postoperative GIB risk. The dataset was divided into training and validation sets in a 7:3 ratio. Predictive performance was evaluated and compared using Receiver Operating Characteristic (ROC) curves and DeLong tests. Calibration curves and decision curve analysis (DCA) were used to assess model calibration and clinical utility. The SHapley Additive exPlanation (SHAP) algorithm was applied for interpretability analysis. This study adhered to the “Transparent Reporting of a Multivariable Prediction Model for Individual Prognosis or Diagnosis + Artificial Intelligence (TRIPOD+AI) guidelines.”

**Results:**

A total of 525 TAAD patients were included, with 63 (12%) developing GIB. Nine predictors were selected via LASSO regression for model construction. The RF model outperformed the SVM, KNN, and DT models in predicting postoperative GIB, with areas under the ROC curve (AUC) of 0.933, 0.892, 0.902, and 0.768, respectively, showing statistically significant differences (DeLong test, *P* < 0.05). Calibration curves and DCA further confirmed the RF model’s excellent calibration and clinical utility. SHAP analysis identified the three most influential clinical features on the RF model’s output: duration of mechanical ventilation (MV), Time to aortic occlusion, and red blood cell (RBC) transfusion.

**Conclusion:**

The machine learning-based predictive model effectively assesses postoperative GIB risk in TAAD patients, aiding healthcare providers in early identification of risk factors and implementation of targeted preventive strategies.

## 1 Introduction

Type A aortic dissection is a severe cardiovascular emergency with a mortality rate as high as 58% (1). Surgical intervention under cardiopulmonary bypass (CPB) is the primary treatment method (2). CPB is a critical life-support technology commonly used in cardiovascular surgeries (3). However, TAAD surgery under CPB often leads to various complications, such as respiratory failure, sepsis, dialysis-dependent renal failure, and gastrointestinal injury (4). Epidemiological studies have shown that the incidence of gastrointestinal injury in TAAD patients after surgery can reach up to 50%, with GIB occurring in 35.0%–64.7% of cases, higher than other gastrointestinal complications (approximately 14.0%) and with the highest mortality rate among them (5–7). The mechanism of postoperative GIB in these patients is complex, involving factors such as hypothermic circulatory arrest, coagulation disorders, and inflammatory responses. Additionally, GIB often has an insidious onset and can easily be mistaken for other abdominal complications in clinical practice (8). This not only increases patients’ postoperative risk and mortality but also poses significant challenges for diagnosis and care after surgery. Therefore, accurately predicting the risk of postoperative GIB in TAAD patients is crucial for optimizing treatment plans and informing clinical decision-making. Although studies have explored the risk factors for postoperative GIB in TAAD patients, there is no current consensus (9–11). Furthermore, existing predictive models have primarily relied on single algorithms, and the accuracy and reliability of their predictions remain to be further validated.

In recent years, with the widespread application of technologies like big data and artificial intelligence in the medical field, ML models have demonstrated superior performance over traditional methods in health assessments of patients with severe conditions and extensive, complex clinical data (12). With their ability to efficiently process large datasets and fast computational speed, ML models have been widely used in disease diagnosis and prognosis evaluation (13–15). However, while ML models can provide accurate predictions, their lack of intuitive interpretability poses challenges and difficulties in practical applications. In 2020, Lundberg et al. (16) developed the SHAP algorithm to explain the outputs of ML models. This algorithm not only reflects the impact of variables on the model based on the positive or negative nature of SHAP values but also quantifies the contribution of each variable in the model through SHAP values, thus addressing the interpretability challenges of ML models.

Therefore, this study comprehensively explores the factors influencing postoperative GIB in TAAD patients from various perspectives, including patient demographics, preoperative medication and blood test indicators, Intraoperative manipulation-related factors, postoperative conditions, and outcome indicators. Additionally, four ML models were developed to predict postoperative GIB in TAAD patients. After screening, the SHAP algorithm was applied to interpret the optimal model. This approach aims to help medical professionals identify the potential risks of postoperative GIB in these patients at an early stage, enabling the implementation of precise preventive strategies. It holds significant value in improving patient outcomes and enhancing healthcare providers’ ability to manage potential risk events.

## 2 Materials and methods

### 2.1 Study population

**FIGURE 1:**
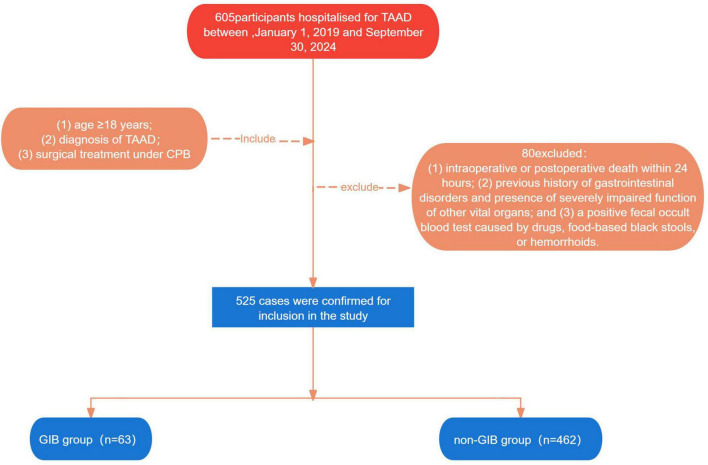
Chart of the specific study population enrollment.

This study retrospectively collected and analyzed the medical records of all patients who underwent TAAD surgery from January 2019 to September 2024 at Shanxi Bethune Hospital. Inclusion criteria: (1) Age ≥ 18 years; (2) Diagnosis of TAAD confirmed by computed tomography aortography; (3) Surgical treatment under CPB. Exclusion criteria: (1) intra-operative or postoperative death within 24 h; (2) history of gastrointestinal disorders and presence of severely impaired function of other vital organs; and (3) a positive fecal occult blood test caused by drugs, food-based black stools, or hemorrhoids. Based on the inclusion and exclusion criteria, 525 patients were finally identified for model development. The patients were categorized into GIB and non-GIB groups according to the presence or absence of GIB after surgery. The retrospective study was approved by the Ethics Committee of Shanxi Bethune Hospital (Approval No. YXLL-2023-283) and was managed in accordance with the Declaration of Helsinki. As this is a retrospective study, it was not registered as a clinical trial. However, patient data underwent compliant de-identification procedures, utilizing only anonymized information to fully safeguard patient privacy. The flow chart of the specific study population enrollment is shown in Figure 1.

### 2.2 Definition

Gastrointestinal bleeding includes upper and lower GIB, with upper GIB occurring in the esophagus, stomach, or duodenum and lower GIB occurring in the small intestine, colon, rectum, or anus, and its clinical manifestations vary depending on the severity and location of the bleeding (17). GIB was defined as meeting all the following criteria: (1) positive fecal occult blood test; (2) clinical manifestations accompanied by hemorrhage (e.g., dizziness, panic attacks, cold sweats, malaise, dry mouth, etc., and, in severe cases, fainting, cold extremities, low urine output, restlessness, shock, etc.); and (3) unexplained progressive decrease in hemoglobin.

### 2.3 Data collection

This study strictly followed the (C*ollection Form for Influencing Factors of Postoperative GIB in TAAD Patients*), which was developed by the research team based on previous literature analysis and expert consultation to collect patient case data. The content includes five sections: basic patient information [age, gender, smoking history, drinking history, body surface area (BSA), body mass index (BMI), Coronary artery disease, hypertension, Cerebrovascular disease, and diabetes]; preoperative medication and blood test indicators [left ventricular ejection fraction (LVEF), glomerular filtration rate (GFR), use of vasoactive drugs, and anticoagulants]; intraoperative factors [Time to CPB, Time to aortic occlusion, deep hypothermic cardiopulmonary arrest (DHCA), Hemorrhage, RBC transfusion, plasma transfusion volume, platelet transfusion volume, Cold precipitation infusion volume]; postoperative conditions [3 days average blood pressure, international normalized ratio (INR), intra-aortic balloon pump (IABP), external temporary pacemaker, continuous renal replacement therapy (CRRT), MV duration, sedative and analgesic drugs, Time of first meal, Time to first ambulation, left ventricular ejection fraction, Low Cardiac Output Syndrome (LCOS), Length of ICU stay, and hospital days]; and outcome indicators (GIB). The data used in this study were retrospectively obtained from the electronic medical record system of this hospital. Sample size calculation adhered to the events per variable (EPV) rule.

### 2.4 Data processing

Variables with missing data exceeding 30% were excluded. For the remaining missing variables, imputation was performed using the MissForest package in R 4.3.3 to ensure the completeness and accuracy of the dataset.

### 2.5 Quality control

This study adopted a staged operational workflow to ensure the independence of data processing, with data entry, de-identification, and analysis performed by separate researchers. The specific implementation steps were as follows: (1) Data collection was independently conducted by two researchers who underwent standardized training and passed competency assessments, using a unified electronic form for dual-track recording; (2) After data collection, dual verification was performed by two individuals to minimize entry errors; (3) Following data entry, 20% of the data were randomly selected for re-verification to ensure accuracy and validity; (4) After anonymization by another researcher, the dataset was transferred to an independent data analyst for processing. Also the TRIPOD checklist was used in this study to assess model transparency (see Supplementary Table 1).

### 2.6 Data analysis and model development

This study utilized SPSS 25.0 for data processing and R 4.3.3 for predictor screening, model construction, and model evaluation. Continuous variables were assessed for normality using the Shapiro-Wilk test. As the continuous variables did not follow a normal distribution, they were expressed as medians and compared between groups using the Mann-Whitney U test. Categorical variables were presented as frequencies or percentages (%) and evaluated for intergroup differences using Pearson’s chi-square test. LASSO regression was applied to further screen potential predictors, and four risk prediction models—RF, SVM, KNN, and DT—were constructed based on machine learning. To address class imbalance in the dependent variable, an undersampling method was employed to resample the data for balance. During model construction, 70% of the sample data were allocated as the training set, with the remaining 30% serving as an independent validation set. A total of 5-fold cross-validation was used for model training, and hyperparameters were tuned via grid search. The predictive performance of the models was evaluated using the area under the ROC curve (AUC), and differences in predictive performance among the four models were compared using the DeLong test, with *P* < 0.05 considered statistically significant. Calibration curves and DCA were analyzed to assess model calibration and clinical utility.

### 2.7 Interpretability analysis

The SHAP algorithm is a versatile ML interpretability method. To further explain the impact and contribution of each feature variable on the final model, the SHAP algorithm provides an explanation value for each feature, representing the extent of its influence on the model’s prediction results. Computed results not only explain the importance of features for individual predictions but can also be used to interpret the feature importance distribution across the entire dataset. Additionally, this method can visually display the influence of each feature on individual data points as well as the importance distribution for the entire dataset. As such, it has become one of the most important interpretability methods in the field of ML and is widely applied across various domains. In this study, R 4.3.3 was used to construct the model, and the SHAP algorithm was employed to generate summary plots and dependency plots to interpret the model.

## 3 Results

### 3.1 Patient characteristics

A total of 525 patients were included in this study for model development, with 63 patients (12%) developing GIB. Grouped analysis was performed based on the occurrence of GIB. Table 1 presents the baseline characteristics of all patients in the GIB and non-GIB groups. Compared to the non-GIB group, the GIB group showed significant differences in age, gender, history of stroke, GFR, Time to aortic occlusion, blood loss, RBC transfusion, plasma transfusion volume, IABP, external temporary pacemaker, CRRT, MV duration, the number of sedative and analgesic drugs used, time to first oral intake, Time to first ambulation, LVEF, LCOS, and ICU length of stay.

**TABLE 1:** Comparison of baseline characteristics between the gastrointestinal bleeding (GIB) and non-gastrointestinal bleeding (GIB) groups.

Characteristics	Non-GIB group (*n* = 462)	GIB group (*n* = 63)	χ^2^/Z value	*P*-value
Age (years)	50 (43–59.75)	55 (45–60.5)	1.99	0.046*
**Gender [n (%)]**				
Female	135 (29 22)	27 (42.86)	4.21	0.040*
Male	327 (70.78)	36 (57.14)		
**Smoking history [n (%)]**				
No	232 (50.22)	30 (47.62)	0.064	0.801
Yes	230 (49.78)	33 (52.38)		
**Drinking history [n (%)]**				
No	230 (49.78)	33 (52.38)	0.064	0.801
Yes	232 (50.22)	30 (47.62)		
BSA (m^2^)	1.81 (1.69–1.958)	1.84 (1.7–1.98)	0.52	0.605
**BMI [kg/m^2^, n (%)]**				
≤ 18.5	35 (7.58%)	3 (4.76%)	4.805	0.307
18.5 < BMI ≤ 24	160 (34.63%)	24 (38.1%)		
24 < BMI ≤ 28	174 (37.66%)	18 (28.57%)		
28 < BMI ≤ 32	66 (14.29%)	11 (17.46%)		
> 32	27 (5.84%)	7 (11.11%)		
**Coronary artery disease [n (%)]**				
No	438 (94.81)	57 (90.48)	1.21	0.272
Yes	24 (5.19)	6 (9.52)		
**Hypertension [n (%)]**				
No	242 (52.38)	33 (52.38)	0.00	1.000
Yes	220 (47.62)	30 (47.62)		
**Cerebrovascular disease [n (%)]**				
No	437 (94.59)	53 (84.13)	8.14	0.004*
Yes	25 (5.41)	10 (15.87)		
**Diabetes [n (%)]**				
No	427 (92.42)	57 (90.48)	0.08	0.772
Yes	35 (7.58)	6 (9.52)		
Preoperative LVEF (%)	56 (49–60)	58 (53–61)	1.02	0.306
GFR [ml/(min⋅1.73 m^2^)]	71.23 (56.52–86.84)	53.57 (35.945–81.15)	3.43	0.001*
**Vasoactive drug [n (%)]**				
No	443 (95.89)	57 (90.48)	2.49	0.115
Yes	19 (4.11)	6 (9.52)		
**Anticoagulant [n (%)]**				
No	347 (75.11)	54 (85.71)	2.89	0.089
Yes	115 (24.89)	9 (14.29)		
Time to CPB (min)	204.5 (171–229.75)	208 (191.5–236)	1.89	0.060
Time to aortic occlusion (min)	124 (106–138)	139 (119–154.5)	5.04	< 0.01*
**DHCA [n (%)]**				
No	98 (21.21)	7 (11.11)	2.93	0.087
Yes	364 (78.79)	56 (88.89)		
Hemorrhage (ml)	800 (500–1,200)	1,200 (849.5–1,500)	4.43	< 0.01*
RBC transfusion (U)	4 (2–6)	8 (5.5–9)	5.62	< 0.01*
Plasma transfusion volume (ml)	600 (400–1,000)	1,000 (794–1143.5)	5.63	<0.01*
Platelet transfusion volume (U)	1 (0–2)	1 (0–2)	0.67	0.502
Cold precipitation infusion volume (U)	10 (0–10)	9 (2.5–10)	1.69	0.092
**3 days average blood pressure [n (%)]**				
Abnormality	351 (75.97)	45 (71.43)	0.40	0.529
Normality	111 (24.03)	18 (28.57)		
INR	1.21 (1.15–1.3)	1.21 (1.16–1.295)	0.215	0.830
**IABP [n (%)]**				
No	461 (99.78)	56 (88.89)	36.89	< 0.01*
Yes	1 (0.22)	7 (11.11)		
**External temporary pacemaker [n (%)]**				
No	456 (98.7)	55 (87.3)	23.54	< 0.01*
Yes	6 (1.3)	8 (12.7)		
**CRRT [n (%)]**				
No	446 (96.54)	44 (69.84)	59.28	< 0.01*
Yes	16 (3.46)	19 (30.16)		
MV duration (h)	24 (17–70.75)	100 (38–172.5)	6.30	< 0.01*
**Sedative and analgesic drugs (Number)**				
≤ 3	349 (75.54)	56 (88.89)	4.87	0.027*
> 3	113 (24.46)	7 (11.11)		
Time to first oral intake (d)	3 (2–6)	8 (5–12.5)	6.68	< 0.01*
Time to first ambulation (d)	9 (7–12)	13 (10–15)	4.87	< 0.01*
Postoperative LVEF (%)	61 (58–66)	64 (61–66)	2.27	0.023*
**LCOS [n (%)]**				
No	430 (93.07)	37 (58.73)	63.09	< 0.01*
Yes	32 (6.93)	26 (41.27)		
Length of ICU stay (d)	8 (5–11)	12 (9–14)	5.80	< 0.01*
Hospital days (d)	21 (13.25–30)	20 (16–22.5)	1.55	0.121

Z, Mann–Whitney test; χ^2^, Chi-square test;

*P < 0.05.

### 3.2 Feature variable selection

**FIGURE 2:**
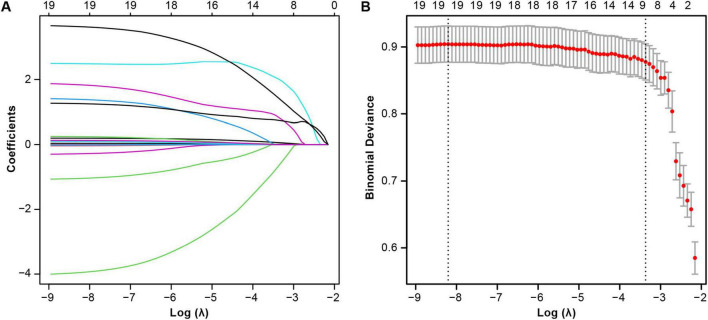
Lasso regression-based variable screening. **(A)** Variation characteristics of variable coefficients. **(B)** The process of selecting the optimal value of the parameter λ in the lasso regression model is carried out by the cross-validation method.

This study incorporated 18 variables showing significant differences in baseline analysis into the LASSO regression model to further screen relevant feature variables. In the LASSO regression model, the lambda value corresponding to the minimum standard error was 0.03, which resulted in nine non-zero coefficient feature variables (Figures 2A, B). These variables were MV duration, Time to aortic occlusion, RBC transfusion, sedative and analgesic drugs, IABP, external temporary pacemaker, CRRT, LCOS, and Length of ICU stay. These variables were included as predictors in the prediction model.

### 3.3 Comparative multi-model analysis

Among the 525 patients, 367 were allocated to the training set and 158 to the testing set. Baseline characteristics between the training and testing sets showed no statistically significant differences (*P* > 0.05) except for postoperative 3 days average blood pressure (see Supplementary Table 2). Models were constructed using 5-fold cross-validation and grid search for hyperparameter tuning (specific parameters are detailed in Supplementary Table 3). Comprehensive evaluation of the four machine learning models (RF, SVM, KNN, and DT) demonstrated that the RF model exhibited the best performance, with DeLong test *P* < 0.05 (see Table 2).

**TABLE 2:** Performance comparison of four machine learning models in predicting postoperative gastrointestinal bleeding (GIB) risk in Type A aortic dissection (TAAD) patients.

Model	AUC (95% CI)	Sensitivity (95% CI)	Specificity (95% CI)	Accuracy (95% CI)	Precision (95% CI)	F1-score (95% CI)	*P*_DeLong test_
RF	0.933 (0.840–0.997)	0.789 (0.597–0.967)	0.971 (0.944–0.998)	0.950 (0.915–0.983)	0.789 (0.595–0.972)	0.790 (0.631–0.926)	–
SVM	0.892 (0.754–0.983)	0.684 (0.457–0.903)	0.978 (0.954–0.993)	0.943 (0.907–0.979)	0.812 (0.599–0.897)	0.743 (0.552–0.913)	0.011*
KNN	0.902 (0.802–0.993)	0.789 (0.59–0.975)	0.964 (0.933–0.996)	0.943 (0.907–0.979)	0.750 (0.547–0.952)	0.769 (0.602–0.919)	0.023*
DT	0.768 (0.648–0.888)	0.526 (0.296–0.753)	0.992 (0.978–0.998)	0.937 (0.899–0.975)	0.909 (0.726–0.987)	0.667 (0.455–0.860)	0.004*

*P < 0.05.

**FIGURE 3:**
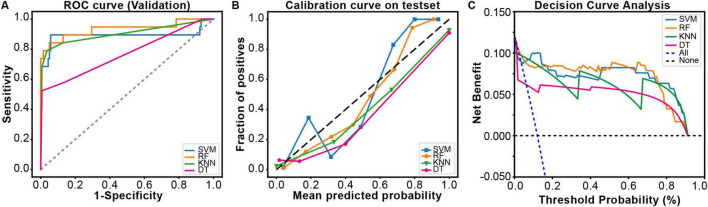
The comprehensive analysis of four ML models. **(A)** The Receiver Operating Characteristic (ROC) curve and area under the curve of the validation set. **(B)** The calibration curve plot of the models. In the calibration curve, the x-axis represents the average predicted probability, and the y-axis represents the actual probability of a positive event occurring. The diagonal line represents the perfectly calibrated reference line. The solid lines of different colors correspond to the fitting lines of the respective models. **(C)** The decision curve analysis (DCA) of the validation set.

Receiver Operating Characteristic curves were plotted using the ggplot2 package in R software (Figure 3A). Compared to the other three models, the RF model (AUC: 0.933, 95% CI: 0.840–0.997) demonstrated significantly higher predictive performance for gastrointestinal bleeding following aortic dissection surgery than the KNN model (0.901, 95% CI: 0.802–0.993), SVM model (0.891, 95% CI: 0.754–0.983), and DT model (0.768, 95% CI: 0.648–0.888). To further evaluate the strengths, limitations, and clinical utility of the models, calibration curves (Figure 3B) and decision curve analysis (Figure 3C) were generated using the rms and rmda packages in R software, respectively, for comprehensive model assessment. The calibration curve revealed that the RF model exhibited lower calibration deviation (0.151) compared to the SVM (0.257), KNN (0.172), and DT (0.153) models, indicating superior predictive accuracy. Decision curve analysis demonstrated favorable clinical applicability of the RF model. In summary, the results of this study indicate that the RF model exhibits robust stability and is the optimal predictive model. Consequently, the RF model was selected for further predictive analysis. All R packages utilized in this study are freely available through the Comprehensive R Archive Network (CRAN).

### 3.4 Model explanation

**FIGURE 4:**
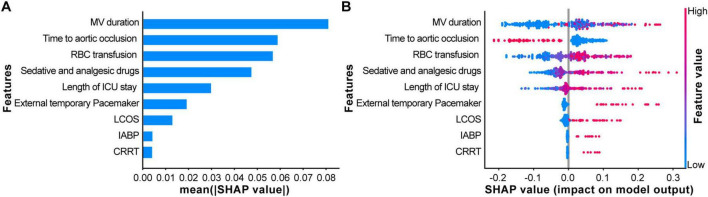
Explaining the model using the SHapley Additive exPlanation (SHAP) analysis. **(A)** The scatter plot of feature distributions using the SHAP analysis. **(B)** Ranking feature importance based on the absolute mean values of SHAP values. [SHAP values represent the predictive features of individual patients and the contribution of each feature to predicting gastrointestinal bleeding (GIB)].

Using the SHAP algorithm, an interpretability analysis was performed to identify the key risk factors influencing postoperative GIB in TAAD patients, providing a visual representation of the proportional differences among variables. The results indicated that the top three most important clinical features influencing the output of the optimal model ranked by importance were MV duration, Time to aortic occlusion, and RBC transfusion (Figure 4).

**FIGURE 5:**
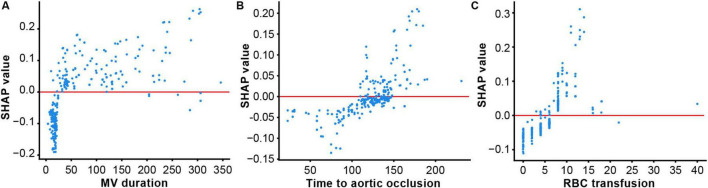
SHapley Additive exPlanation (SHAP) dependency plot for the top six clinical features contributing to GBDT model. **(A)** Mechanical ventilation (MV) duration; **(B)** Time to aortic occlusion; **(C)** Red blood cell (RBC) transfusion. SHAP values for specific features exceed zero, representing an increased risk of gastrointestinal bleeding (GIB).

Based on the SHAP summary plot, SHAP Dependency Plots were further generated for the top three clinical features to explain their impact on GIB in patients. In the dependency plots, the vertical axis represents the SHAP value of the clinical feature, while the horizontal axis represents the range of variation for that feature. A SHAP value greater than zero indicates an increased risk of postoperative GIB for these patients (Figure 5).

## 4 Discussion

### 4.1 ML predictive model construction and evaluation

Machine learning algorithms enable deep exploration of data to analyze intrinsic relationships within datasets, demonstrating distinct advantages in big data processing (18, 19). However, although deep learning and more advanced ensemble learning methods may optimize model performance when handling complex data, we prioritized classical ML models due to the limitations of our dataset characteristics and the high demands of clinical real-time decision-making. Consequently, we successfully trained and validated four ML algorithm-based models—RF, SVM, KNN, and DT—to assess the risk of postoperative GIB in TAAD patients. Furthermore, the practicality and accuracy of the four models were evaluated using ROC curves, calibration curves, DCA, and metrics such as sensitivity, specificity, and F1 scores. Results revealed that the RF model exhibited strong clinical utility, high predictive accuracy, and optimal comprehensive performance.

Random Forest is an ensemble learning algorithm based on decision trees, which constructs models by training multiple decision trees and employs voting mechanisms or averaging methods to derive final predictions. Due to its outstanding performance in feature extraction, overfitting resistance, and data noise reduction (20), the RF algorithm is characterized by strong practicality and high accuracy. Wallace et al. (21) utilized the RF algorithm, integrating 500 decision trees and evaluating the importance of 157 features during training, to comprehensively predict risk factors for stroke events during nocturnal sleep, significantly enhancing the model’s clinical applicability. Additionally, multiple studies in postoperative cardiac prediction (22–24) have demonstrated that prediction models constructed using the RF algorithm achieve significantly higher accuracy compared to other ML algorithms such as logistic regression, SVM, and KNN. Therefore, the advantages of the RF algorithm in data processing and feature selection fully satisfy the requirements for practicality and accuracy in predicting GIB risk in TAAD patients.

However, although the RF model demonstrated high predictive performance (AUC = 0.933) and clinical utility in this study, its clinical application remains constrained by the inherent limitations of retrospective research, such as selection bias and the absence of critical dynamic physiological parameters (e.g., real-time blood pressure, dynamic coagulation function changes) (25). Prospective studies, leveraging rigorous enrollment design, real-time data acquisition, and dynamic monitoring, can not only effectively control confounding factors but also enable continuous iterative optimization of the model based on newly acquired continuous clinical data. This approach would validate its robustness in dynamic clinical environments (e.g., variations in surgical practices, adjustments in treatment strategies) and assess its generalizability across diverse medical centers and patient populations. Therefore, further validation of this model through prospective studies remains necessary.

### 4.2 Interpretability of ML models

Chinese TAAD patients exhibit an average onset age 10–20 years younger than their Western counterparts, predominantly affecting young and middle-aged adults (26), resulting in longer life expectancy. During initial surgical interventions, particular emphasis must be placed on long-term outcomes to minimize or avoid secondary reinterventions. This distinctiveness is reflected not only in the younger age of onset but also in the critical impact of postoperative complications—particularly GIB—on patients’ long-term quality of life and survival. To effectively evaluate patient prognosis and optimize treatment strategies, innovation in clinical decision-making tools is imperative. However, while ML predictive models serve as effective tools for clinical disease diagnosis and prognostic assessment, their inherent “black box” nature complicates clinicians’ understanding of model mechanisms, thereby limiting clinical adoption. To address this, we employed the SHAP algorithm to analyze the interpretability of the RF model for predicting postoperative GIB risk in TAAD patients. Results identified three clinically influential features: MV duration, Time to aortic occlusion, and volume of RBC transfusion, which serve as critical indicators for predicting postoperative GIB in this population. This approach not only enhanced model transparency but also provided more precise and reliable evidence to inform clinical decision-making.

Postoperative TAAD patients require MV to ensure respiratory function and improve oxygenation due to surgical trauma, residual anesthetic effects, pain, and unstable vital signs. This study demonstrated that TAAD patients with MV duration exceeding 24 h postoperatively face a significantly higher risk of GIB (Figure 5A), a finding consistent with multiple studies (27, 28). However, a meta-analysis (29) reported no conclusive evidence linking MV to GIB risk in critically ill patients. This discrepancy may arise from differences in study populations, as the meta-analysis focused on ICU patients, whereas our cohort comprised post-cardiac surgery patients. Additionally, population heterogeneity likely contributes to variations in outcomes regarding MV’s impact on GIB. Thus, the relationship between MV duration and GIB risk warrants further investigation. Nevertheless, for patients requiring MV beyond 24 h, optimized ventilator management strategies—including precise ventilator weaning assessment, early respiratory muscle training, and concurrent monitoring of gastric mucosal pH during MV—should be implemented to mitigate risks. These measures aim to balance respiratory support with minimizing potential adverse outcomes.

Aortic occlusion is a necessary procedure during TAAD surgery under CPB, and its duration critically influences surgical outcomes and postoperative recovery. Yang et al. (30) demonstrated a positive correlation between intraoperative aortic occlusion time and postoperative GIB risk. The SHAP dependence plot in this study confirmed that when aortic occlusion time exceeds 100 min, TAAD patients face a significantly elevated risk of postoperative GIB (Figure 5B), further validating the importance of aortic occlusion time in predicting GIB risk after TAAD surgery. Prolonged aortic occlusion time reflects extended CPB duration, during which surgical trauma, intraoperative hypothermia, and non-pulsatile perfusion contribute substantially to systemic complications. Therefore, optimizing surgical techniques to precisely control aortic occlusion time or implementing specific myocardial protection strategies during the procedure is crucial to minimizing the adverse effects of CPB. Additionally, close postoperative monitoring of microcirculatory status and dynamic adjustment of vasoactive medications based on intestinal perfusion pressure may help reduce complication rates.

Type A aortic dissection surgery is often associated with significant intraoperative blood loss, necessitating RBC transfusion to maintain hemodynamic stability and adequate blood volume. However, multiple studies (11, 31) have shown that excessive RBC transfusion in cardiac surgery may trigger transfusion-related inflammatory responses and storage lesions due to prolonged RBC preservation, both of which increase postoperative GIB risk. This study further corroborates these findings, demonstrating that a volume of RBC transfusion exceeding 4 U during surgery significantly elevates the risk of postoperative GIB in TAAD patients, highlighting the critical importance of maintaining appropriate intraoperative RBC transfusion volumes to reduce GIB incidence (Figure 5C). Therefore, clinicians should adopt strict transfusion thresholds, closely monitor postoperative free hemoglobin levels, and implement plasma exchange when necessary to systematically mitigate transfusion-related complication risks.

### 4.3 Study limitations

(1) Clinical feature data were retrospectively collected, which may introduce information bias. (2) This study included postoperative TAAD patients but did not perform stratified analyses based on different surgical techniques, nor did it explore the specific impact of surgical technique differences on GIB. Further research is needed to examine the relationship between different surgical techniques and postoperative GIB in patients. (3) Shanxi Bethune Hospital, as a national regional medical center construction Project, has its cardiac surgery department recognized as a provincial key clinical specialty in Shanxi Province. This institutional context renders the cohort of TAAD patients treated at this hospital both typical and representative. However, our study is currently limited to patients treated at Shanxi Bethune Hospital. Consequently, the predictive model constructed based on this specific patient population may demonstrate optimal performance when applied to Han Chinese in north china regions sharing similar demographic characteristics and healthcare resource allocation patterns as Shanxi Province. Future studies will involve multicenter collaborations to expand the sample size by incorporating ethnically and geographically diverse patient populations, thereby validating and enhancing the model’s external validity.

## 5 Conclusion

The RF model is more advantageous in predicting the risk of postoperative GIB in patients with TAAD, and the three clinical characteristics that have a greater impact on the model are MV duration, the time of aortic occlusion, and the amount of intraoperative RBC transfusion The results of the above study help to increase the understanding of the ML model among clinical healthcare professionals, and promote the clinical application of the model, so that we can identify the population with a high risk of postoperative GIB after TAAD in the early stage and optimize treatment regimens and make clinical decisions in the best interest of patients.

## Data Availability

The original contributions presented in this study are included in this article/Supplementary material, further inquiries can be directed to the corresponding author.
